# Supplementary material for: Programmable nanowrinkle-induced room-temperature exciton localization in monolayer WSe2
Source: Nat Commun. 2024 Feb 20;15:1543. doi: 10.1038/s41467-024-45936-2 (PMC10879107; doi:10.1038/s41467-024-45936-2)
Supplement: Supplementary file 1 — Supplementary Information [file 41467_2024_45936_MOESM1_ESM.pdf]

Supporting Information to Programmable Nanowrinkle-Induced Room-Temperature Exciton Localization in Monolayer WSe_2

Emanuil S. Yanev[1], Thomas P. Darlington[1], Sophia A. Ladyzhets[1], Matthew C. Strasbourg[2], Chiara Trovatello[1], Song Liu[1], Daniel A. Rhodes[1,3], Kobi Hall[1], Aditya Sinha[1], Nicholas J. Borys[2*], James C. Hone[1*], and P. James Schuck[1*]

[1]Department of Mechanical Engineering, Columbia University, New York, NY, USA

[2]Department of Physics, Montana State University, Bozeman, MT, USA

[3]Department of Materials Science and Engineering, University of Wisconsin-Madison, Madison, WI, USA

[*]p.j.schuck@columbia.edu, jh2228@columbia.edu, nicholas.borys@montana.edu

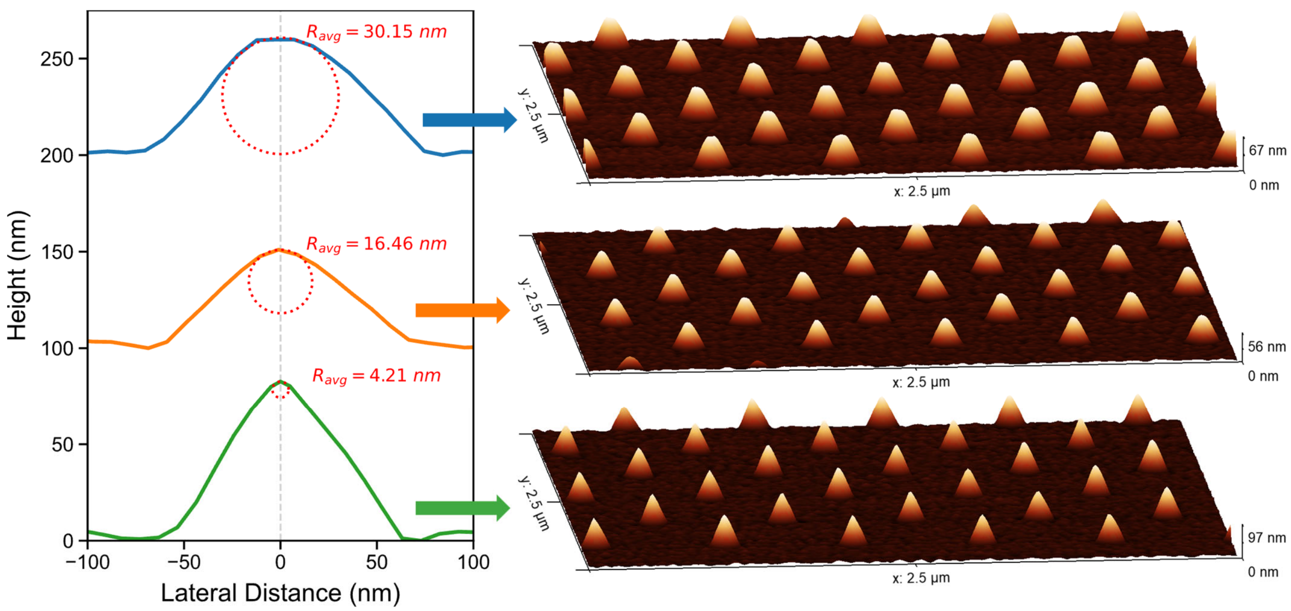

SI Figure 1. Tailored stressor tip radii. Nanocones of varying sharpness can be produced on a single sample. Cross-sections of cones from 3 different regions are shown on the left, with a vertical offset for clarity. The red circles depict the average tip radius for each array, the values of which are extracted from a full three-dimensional fitting of all the cones shown in the corresponding AFM topography maps on the right.

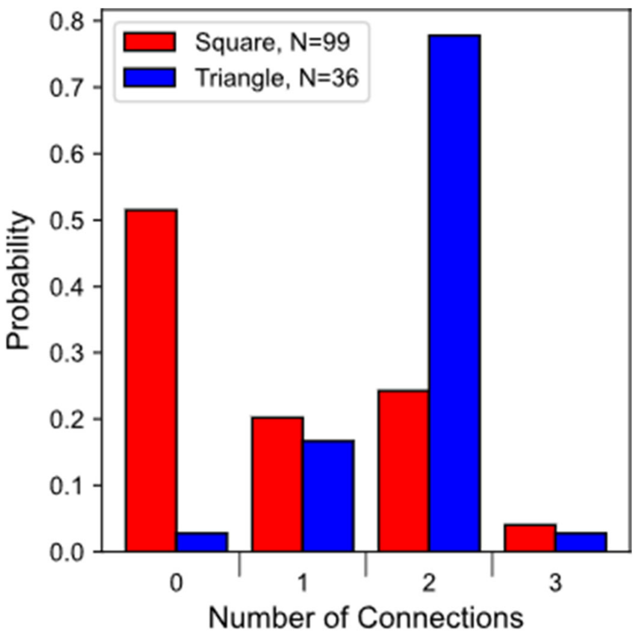

SI Figure 2. Histogram of wrinkle connections between cones for both square and triangular arrays. The probability distributions are clearly dissimilar, which could be due to a multitude of factors including differences in stressor size, residual stresses, and crystallographic alignments.

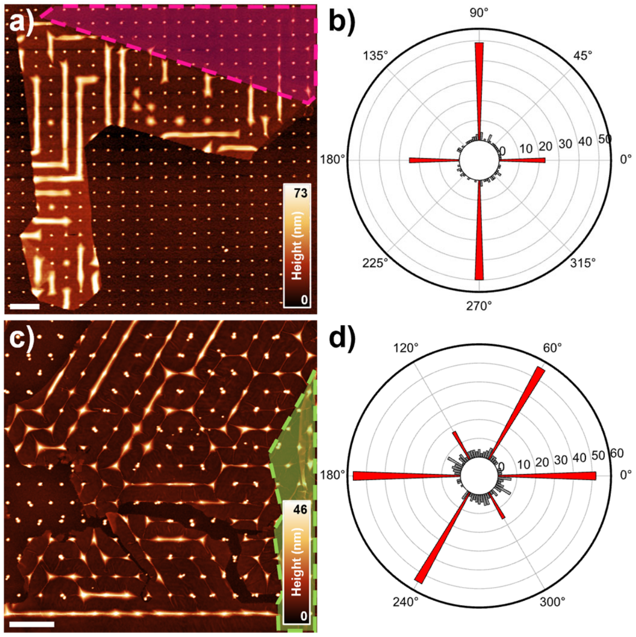

SI Figure 3. Wrinkle formation in thicker regions of WSe_2 adjacent to the monolayers in Fig. 2 of the main text. (a) AFM topography of multilayer WSe_2 on a square lattice of nanocones. The pink shaded area is part of the monolayer, which is the focus of the main text (see Fig. 3 and SI Fig. 6 for a complete view). (b) A polar histogram of wrinkle directions in (a), excluding the shaded region. (c) AFM topography of multilayer WSe_2 on a triangular lattice of nanocones. The green shaded area is the transition zone marked in Fig. 2d of the main text. (d) A polar histogram of wrinkle directions in (c), excluding the shaded region. The bins corresponding to lattice directions are colored red in both histograms. All scale bars are 1 μm.

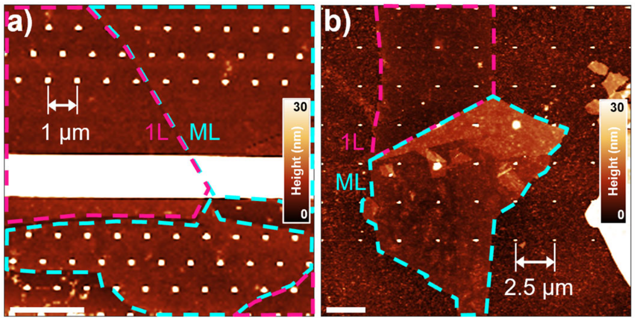

SI Figure 4. Samples with increased array pitch. WSe_2 on (a) 1 μm and (b) 2.5 μm spaced nanocones. The pink dashed boundaries delineate monolayer regions, while the cyan areas are multilayer. The large horizontal stripe in (a) is a gold divider from the nanocone fabrication process. No array wrinkles are evident in these samples, highlighting the fact that lattice spacing is an important control parameter for large wrinkle formation. Further investigation is needed to properly quantify this effect.

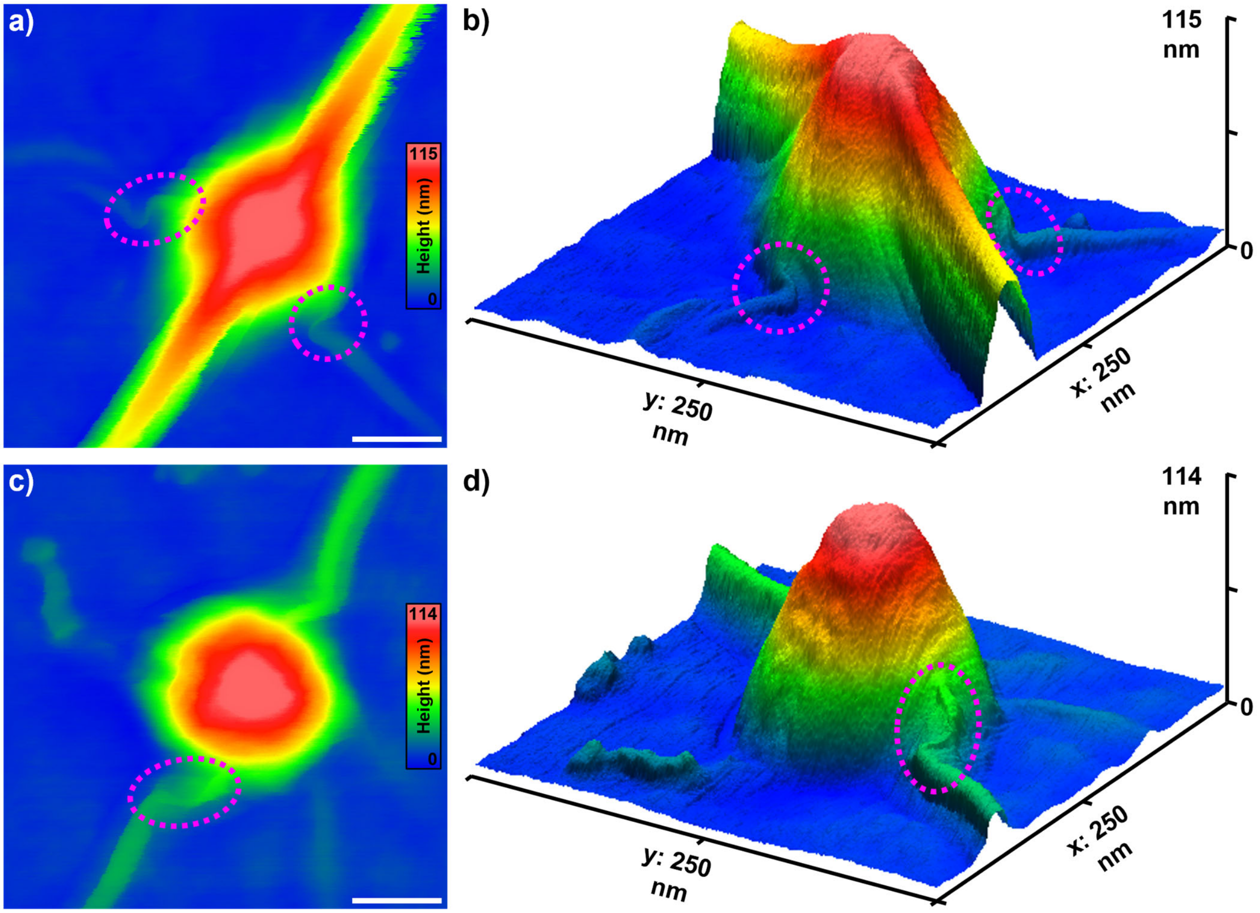

SI Figure 5. High resolution AFM scans of twisted wrinkles in Fig. 2e. Topography of the leftmost nanocone displayed in both 2D (a), and 3D (b). Note the presence of a large array wrinkle, as well as much smaller fine wrinkles. (c) and (d) show similar twisting in small wrinkles around the central nanocone in Fig. 2e. In all four panels, the regions of interest are circled in magenta. The in-plane meandering bends are on the order of 5-10 nm, and vertical cross-sections perpendicular to the wrinkles yield radii even smaller than the reported AFM tip radius—nominally 2 nm for the probes used (Bruker SCANASYST-AIR). All scale bars are 50 nm.

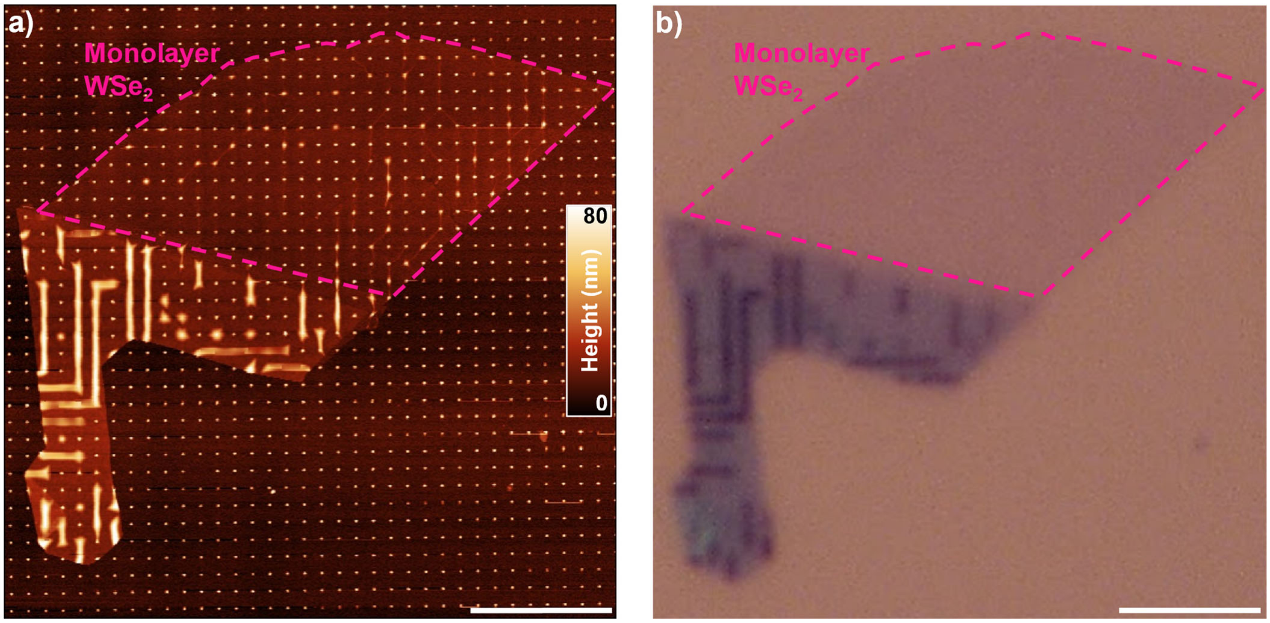

SI Figure 6. Visibility of wrinkles with traditional microscopes. (a) AFM topography and (b) white light optical imaging of WSe_2 on a square nanocone array. The salient features are big vertical and horizontal wrinkles in the multilayer region, which can be seen in both panels, in contrast to the smaller monolayer wrinkles. These highlight the dramatic impact of increased layer thickness on the size of wrinkles formed. All scale bars are 4 µm.

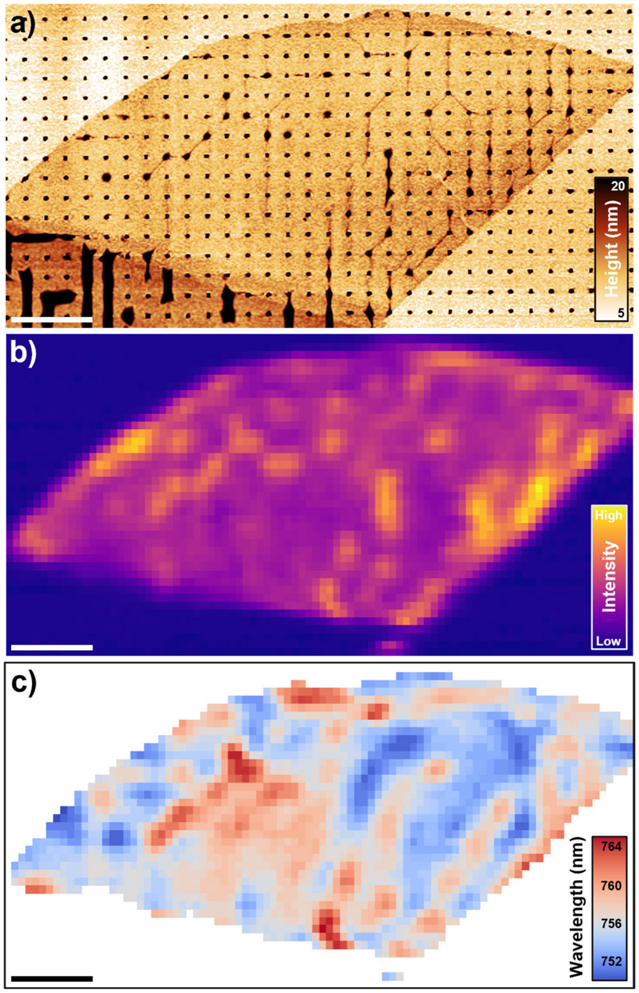

SI Figure 7. Additional details of far-field emission. (a) AFM topography of the monolayer in Fig. 3, reproduced here to aid comparison. (b) Confocal PL map of the total integrated intensity showing spatially inhomogeneous emission. (c) The calculated spectral median for pixels above some threshold in (b). Many similar features can be identified across all three panels, demonstrating a clear link between structure, brightness, and emission energy. All scale bars are 2 µm.

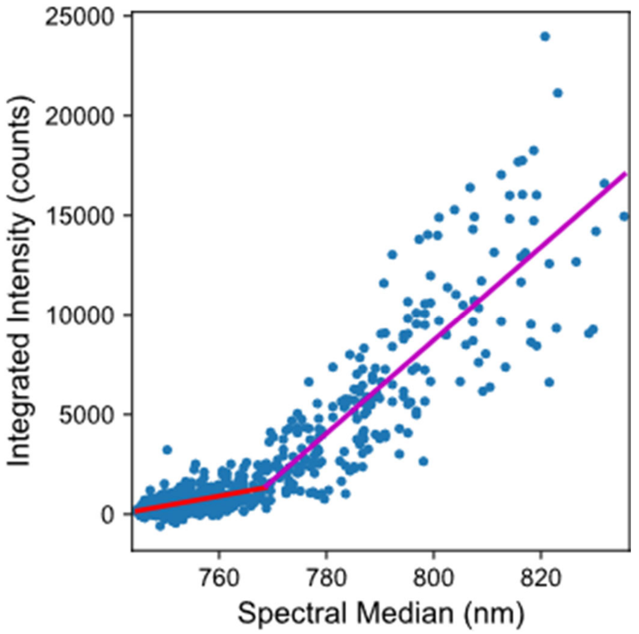

SI Figure 8. Correlation between the spectral median and LX emission intensity in Fig. 5 (panels c and d). The piecewise linear trend indicates the presence of two populations: spectra which lack appreciable localized states are dominated by residual PX emission left over after far-field subtraction and tend to be dim, whereas prominent LX peaks simultaneously redshift the median and increase the overall brightness.

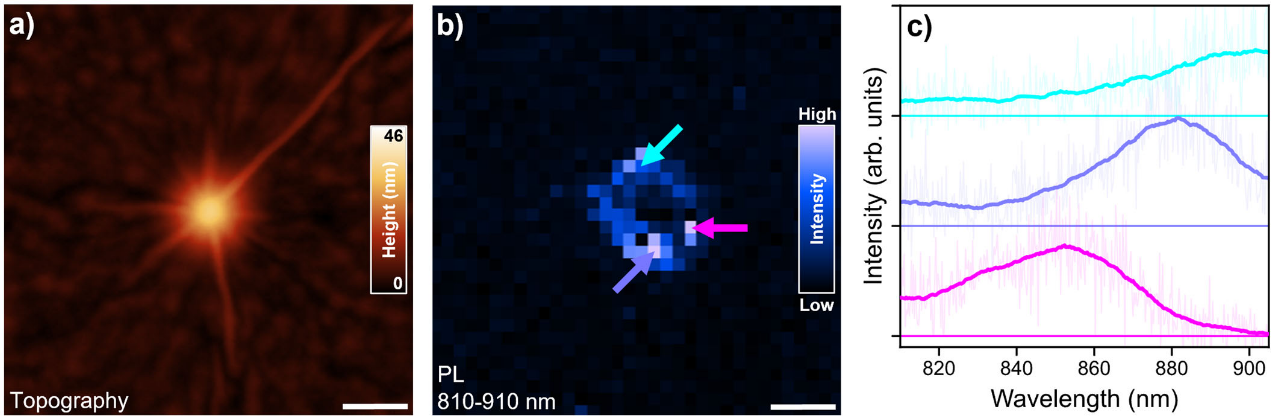

SI Figure 9. Data from an additional cone location. The AFM height map in (a) corresponds to the bottom left cone in Fig. 2b, and shows fine wrinkles radiating out from the center, as well as a larger array wrinkle. (b) Nano-PL mapping of the region reveals a ring of LX emission with a dim center. (c) Several spectra, taken from the locations marked with arrows in (b), highlighting the existence of very red LX states out to at least 900 nm. All scale bars are 100 nm.

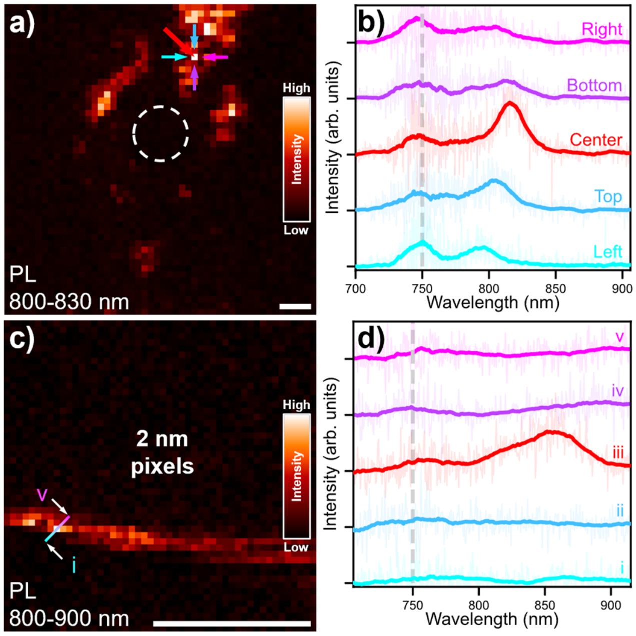

SI Figure 10. Further evidence of exciton localization. (a) The same nano-PL map as in Fig. 5d, only with a narrower integration window and a different location singled out with colored arrows. (b) Spectra from the pixel marked with the red arrow in (a) and its four nearest neighbors, showing localization in multiple directions. (c) A very high-resolution nano-PL map of a wrinkle-like feature on another nanocone sample. Note the change in scale: the line of LX emission would be less than one pixel wide in (a). (d) Spectra from a line-cut through the brightest emission, with the endpoints marked in (c) by arrows and lowercase roman numerals. The LX state at the center of the cut is well confined to a single 2 nm pixel. All scale bars are 50 nm.

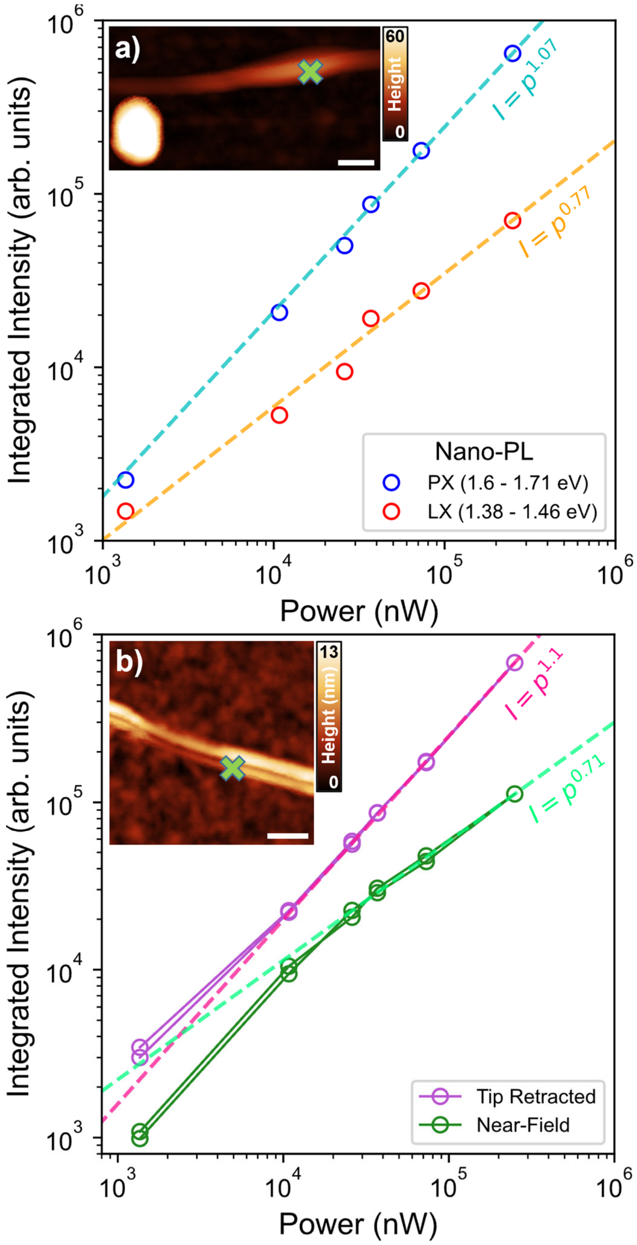

SI Figure 11. Near-field power dependence of localized emitters. (a) Intensity as a function of incident power for integration regions around the primary exciton (725–775 nm, blue circles) and low-energy emitters (850–900 nm, red circles) in an array wrinkle. The tip location during collection is marked with a green "X" on the inset AFM map. The dashed lines are power law fits to the data which show linear (sublinear) scaling for the PX (LX) region. A half decade vertical offset has been applied to the red data points for easier comparison. (b) Total integrated intensity as a function of incident power with the tip in contact (green circles) and out of contact (purple circles) with an array wrinkle. The green "X" on the inset AFM map marks the location of interest. In order to check for drift, the power was swept up and then back down with minimal hysteresis observed from start to finish. Dashed lines are fits to a subset of the data comprising of the 3 highest powers (5 data points). The purple data points have been vertically offset by half a decade for clarity. All scale bars are 100 nm.
